# Low grade mosaic for a complex supernumerary ring chromosome 18 in an adult patient with multiple congenital anomalies

**DOI:** 10.1186/1755-8166-3-13

**Published:** 2010-07-09

**Authors:** Lars T van der Veken, Marianne MJ Dieleman, Hannie Douben, Judith C van de Brug, Raoul van de Graaf, A Jeannette M Hoogeboom, Pino J Poddighe, Annelies de Klein

**Affiliations:** 1 Department of Clinical Genetics, Erasmus University Medical Center, Rotterdam, The Netherlands

## Abstract

**Background:**

Several cases have been reported of patients with a ring chromosome 18 replacing one of the normal chromosomes 18. Less common are patients with a supernumerary ring chromosomes 18. High resolution whole genome examination in patients with multiple congenital abnormalities might reveal cytogenetic abnormalities of an unexpected complexity.

**Results:**

We report a 24 years old male patient with lower spinal anomalies, hypospadia, bifid scrotum, cryptorchism, anal atresia, kidney stones, urethra anomalies, radial dysplasia, and a hypoplastic thumb. Some of the anomalies overlap with the VACTERL association. Chromosome analysis of cultured peripheral blood lymphocytes revealed an additional ring chromosome in 13% of the metaphases. Both parents had a normal karyotype, demonstrating the *de novo *origin of this ring chromosome. FISH analysis using whole chromosome paints showed that the additional chromosomal material was derived from chromosome 18. Chromosome analysis of cultured fibroblasts revealed only one cell with the supernumerary ring chromosome in the 400 analyzed. To characterize the ring chromosome in more detail peripheral blood derived DNA was analyzed using SNP-arrays. The array results indicated a 5 Mb gain of the pericentromeric region of chromosome 18q10-q11.2. FISH analysis using BAC-probes located in the region indicated the presence of 6 signals on the r(18) chromosome. In addition, microsatellite analysis demonstrated that the unique supernumerary ring chromosome was paternally derived and both normal copies showed biparental disomy.

**Conclusions:**

We report on an adult patient with multiple congenital abnormalities who had in 13% of his cells a unique supernumerary ring chromosome 18 that was composed of 6 copies of the 5 Mb gene rich region of 18q11.

## Background

Structural chromosomal abnormalities involving chromosome 18, including del(18q), del(18p) and r(18), are frequently occurring autosomal anomalies which are present in approximately 1/40,000 live births[1-4]. Ring chromosomes 18 have been reported in a significant number of cases. In most cases the ring chromosome 18 replaces a normal maternal or paternal chromosome 18. However, few cases have been described with supernumerary small ring chromosomes 18[3-13]. Timur *et al*. described a patient with a supernumerary r(18) chromosome that contained chromosomal material from 18p in 24% of his cells and presented with Klippel-Trenaunay Syndrome [5]. Callen et al. described a patient with a short stature, normal intelligence and no dysmorphic features who had a supernumerary r(18) chromosome in 85% of the cells[6]. Jenderny *et al*. described a phenotypically and mentally normal women who had a small ring chromosome 18 in 2% of her cells with breakpoints approximately at 18q11 and 18q23[7]. In her daughter the ring chromosome 18 was not present supernumerary and replaced one of the normal chromosomes 18 in all her cells. In our patient a supernumerary ring chromosome 18 was detected in 13% of the metaphases of peripheral blood lymphocytes. The ring chromosome had a unique structural composition, which was different from the supernumerary ring chromosome 18 cases previously described.

## Case presentation

### Clinical details

The patient was born after an uncomplicated pregnancy of 37 weeks as the third son of healthy non-consanguineous parents with a weight of 2670 grams. There was a single umbilical artery. In the first week of life multiple congenital malformations were diagnosed, e.g. anal atresia, dysplastic lumbar and sacral vertebrae, penoscrotal hypospadia, cryptorchism on both sides, bifid scrotum, ureteral flow abnormality (dilatation of the higher urinary tract caused by vesico ureteral reflux, trabeculated bladder), radial dysplasia and thumb hypoplasia on the right upper extremity and preaxial polydactyly of the left hand. The diagnosis VACTERL-association was made, although there were no abnormalities of heart, trachea and esophagus. The urinary flow problems resulted in frequent infections and some episodes of urosepsis, finally causing chronic renal insufficiency. In his teens he underwent cholecystectomy because of an abnormal gallbladder full of stones. A pancreas fissum was seen. Neurologic investigation showed no signs of tethered cord syndrome. School performances were normal, there were no reasons for IQ testing. As an adult his height was on the 3rd centile and his headcircumference was normal.

### Molecular and cytogenetic studies

**Figure 1:**
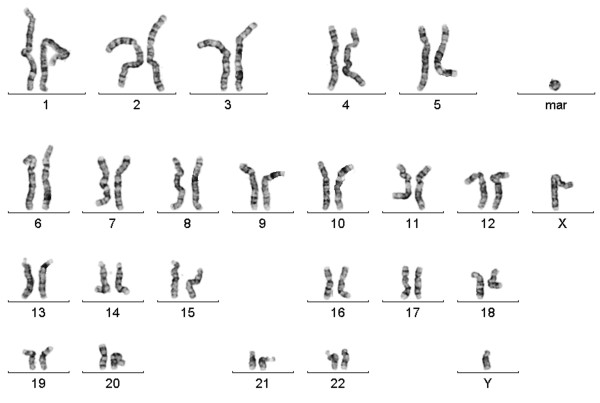
**GTG-banded karyotype from cultured peripheral blood lymphocytes of the patient**. In 13% (4/30) of the metaphases a supernumerary ring chromosome was detected (mar: marker).

Routine karyotyping on GTG banded metaphase spreads (550-band level) from cultured peripheral blood cells revealed an additional ring chromosome in 4 of 30 metaphases (13%) analyzed (Figure 1). The remaining metaphases showed a normal karyotype. Chromosome analysis on GTG banded metaphase spreads (400-band level) of cultured fibroblast cells showed a normal male karyotype (> 30 metaphases analyzed). Karyotyping of the parents showed normal karyotypes.

**Figure 2:**
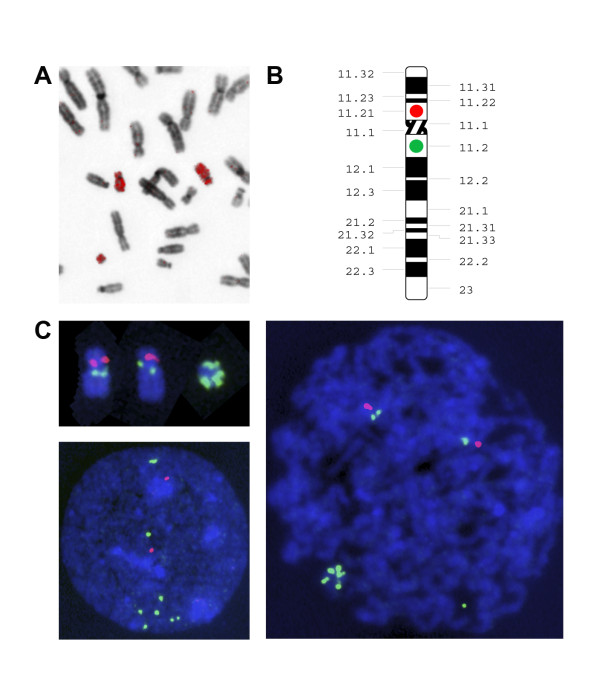
**FISH analysis of cultured peripheral blood lymphocytes of the patient**. **A**. The supernumerary ring chromosome stained positive with whole chromosome paint probes for chromosome 18. **B**. Localization of BAC probes RP11-411B10 (red dot) and RP11-79F3 (green dot) on chromosome 18. **C**. Metaphase and interphase/prometaphase FISH analysis using the BAC probes RP11-411B10 (red) and RP11-79F3 (green) confirmed the presence of the pericentromeric region of 18q11 on the ring chromosome.

**Figure 3:**
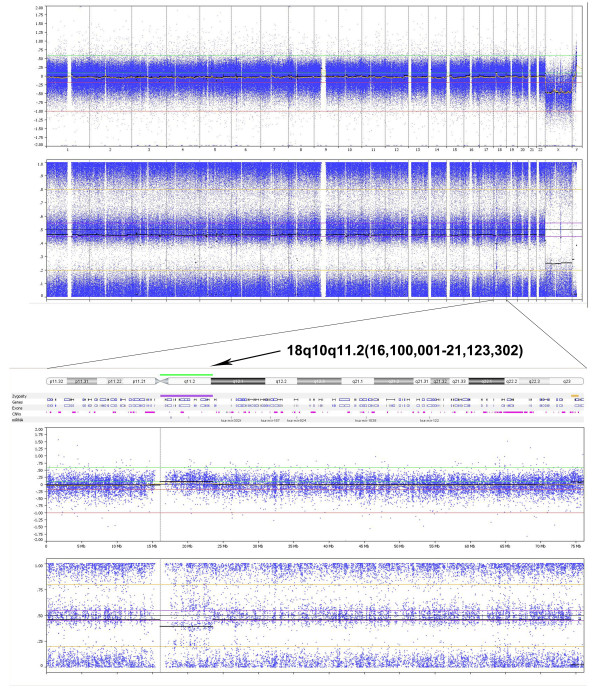
**Whole genome analysis using SNP arrays on peripheral blood derived patient DNA**. An Illumina 610 quad array indicated a ~5 Mb gain of the pericentromeric region of 18q10q11.2(16,100,001-21,123,302). The upper panel shows the array results for the whole genome, while the lower panel shows the results obtained for chromosome 18. The green bar and arrow indicate the amplified region. On the X-axis the chromosomes and chromosomal region are indicated. The upper Y-axis shows the Log2 R ratio and the lower Y-axis indicates the B allele frequency.

Subsequent FISH analysis of peripheral blood cells using whole chromosome paint 18 indicated that the material was derived from chromosome 18 (Figure 2A). To determine the chromosomal breakpoints and to characterize the ring chromosome further whole genome analysis using SNP arrays was performed on peripheral blood derived DNA (Figure 3). Both the Affymetrix 250 K Nsp1 SNP-array and the Illumina 610 quad array confirmed that the additional chromosomal material was derived from chromosome 18 and indicated a ~5 Mb gain of the pericentromeric region of chromosome 18q10q11.2(16,100,001-21,123,302). Metaphase and interphase FISH analysis using BAC-probes from the pericentromeric region of chromosome 18q11 (Figure 2B) showed 6 signals on the r(18) chromosome in 11% of the peripheral blood cells (Figure 2C). Although all detected ring chromosomes 18 showed 6 signals of the pericentromeric BAC-probes, it cannot be excluded that related ring chromosomes 18 with a different structural organization were present but that the low grade mosaicism prevented their detection. FISH analysis of cultured fibroblast cells only showed 1 cell with the supernumerary ring chromosome in 400 cells.

**Figure 4:**
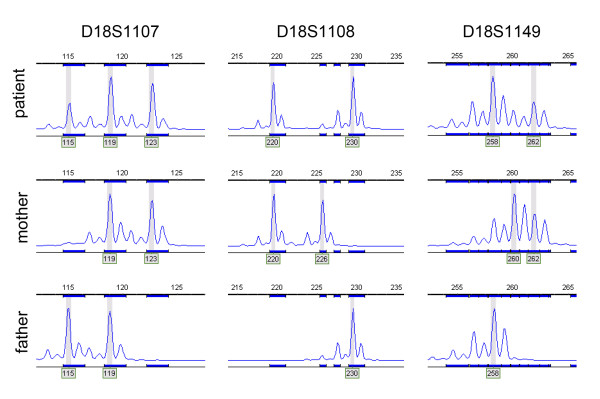
**Microsatellite analysis for the identification of the parental origin of the supernumerary ring chromosome**. Genescan analysis on a panel of informative microsatellite markers located in the pericentromeric region of chromosome 18 indicated that the ring chromosome was paternally derived and that both normal copies of chromosome 18 showed biparental disomy. Informative microsatellite markers were: D18S1107, D18S1108 and D18S1149.

To determine the parental origin of the additional ring chromosome, informative microsatellite markers within the 5 Mb pericentromeric region on chromosome 18 were selected. Genescan analysis of microsatellite marker D18S1107 on patient DNA showed that the patient possessed three different alleles of the respective marker (Figure 4). The mosaic presence of the r(18) chromosome lead to a smaller area under the curve of the allele present on the ring chromosome. Since the mother and father shared one microsatellite allele of the marker D18S1107, the genescan results from this marker did not exclude biparental disomy or maternal uniparental heterodisomy of the normal chromosomes 18. However, both the microsatellite marker D18S1108 and D18S1149 showed maternal alleles that were not detected in the patient, which excludes the possibility of maternal uniparental heterodisomy (Figure 4). Thus, the unique supernumerary ring chromosome 18 detected in the patient was paternally derived, while both normal chromosomes 18 in the patient showed biparental disomy. To exclude paternal mosaicism for the ring chromosome 100 GTG banded metaphases of the father were screened additionally for the presence of supernumerary chromosomes or abnormal chromosomes 18. No such abnormal chromosomes were detected in the father. Since both parents had normal karyotypes, the r(18) arose *de novo*. Therefore, the patient's karyotype was described as: 47,XY,+r(18)(q10q11.2)[4].nuc ish 18q11.2(RP11-79F3x8)[30/281]/46,XY[26]dn.

In summary, we detected a unique supernumerary ring chromosome 18 in a male patient with various phenotypic aberrations. In 11-13% of peripheral blood lymphocytes the additional ring chromosome lead to an octasomy of ~5 Mb of the pericentromeric region of chromosome 18.

## Discussion

Small ring marker chromosomes have been shown to originate from the centromere and from the adjacent pericentric regions of a wide variety of chromosomes[6]. Postzygotic formation of the ring chromosome or postzygotic instability resulting in loss during cell division my explain the mosaic state of the ring chromosome. The postzygotic instability can be caused by the instability and dysfunction of the centromere and/or instability of the ring chromosome. Cases with small ring chromosomes frequently show subclones lacking the ring chromosome resulting in mosaicism of ring containing cell lines and monosomic cell lines when the ring chromosome replaces one of the normal chromosomes [8,9]. In the present case we found a paternally derived supernumerary ring chromosome in mosaic with a normal cell line. The mosaic supernumerary presence of the ring chromosome together with the observation of normal parental karyotypes may indicate that the ring was formed during sperm meiosis I, and that by subsequent non-disjunction and postzygotic instability of the ring a low grade mosaic for the r(18) was generated[10]. Alternatively, the r(18) originated from a trisomic zygote generating normal diploid cell lines (trisomic rescue) through mitotic non-disjunction[11,12]. Finally the presence of the supernumerary ring chromosome may be explained by the model proposed by Daniel *et al. *[13]. According to this model, the ring chromosome may originate from an extra haploid pronucleus derived from a superfluous sperm that is usually degraded by DNAses or other means, but accidentally escaped degradation. After transfection into the zygote nucleus small ring chromosomes may occur. The extra haploid pronucleus may be generated by a multiple fertilization or a delayed incorporation into one blastomere of an extra sperm[14].

**Figure 5:**
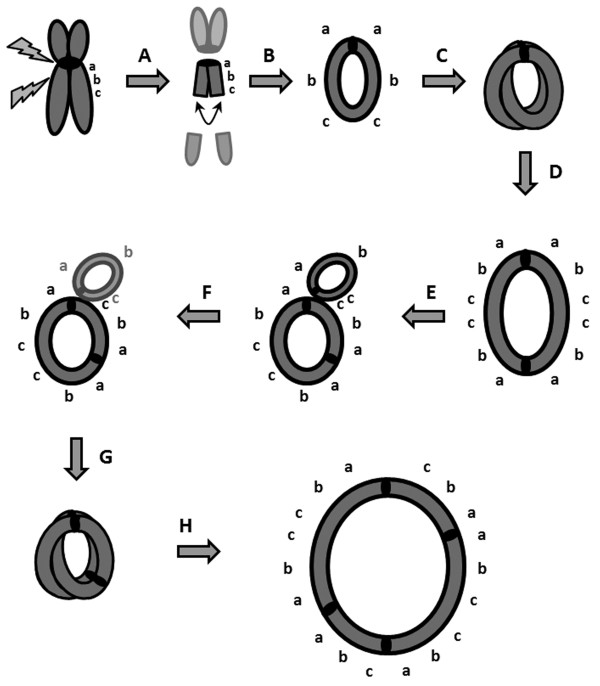
**Putative mechanism for the formation of the tetracentric ring containing 6 copies of the pericentric region of 18q10-10q11.2 A**. A supernumerary chromosome 18 from the father underwent transverse misdivision of the centromere or successive breaks at or adjacent to the centromere and in the long arm of chromosome 18. **B**. A small ring chromosome was generated through repair of the double-strand break of the chromosome by fusion of the two sister chromatids (U-type sister chromatin exchange). **C**. After DNA synthesis a subsequent sister chromatid exchange occurred. **D**. This lead to the formation of a dicentric ring chromosome containing 4 times the pericentric region of 18q10-10q11.2. **E**. By intrastrand homologous recombination and homologous end joining a monocentric ring chromosome and a bicentric ring chromosome (containing 3 times the pericentric region) were generated. **F**. Since most centromeres of the supernumerary ring were likely to be inactivated, the newly formed monocentric ring received one of the inactive centromeres, leading to loss of the monocentric ring in subsequent cell divisions. **G**. Sister chromatid exchange in the bicentric ring (**H**.) resulted in a tetracentric ring containing 6 copies of the pericentric region of 18q10-10q11.2.

Various cases of double-sized ring chromosomes have been described previously [2,15,16]. However, the ring chromosome described in the present case had a unique structural organization consisting of a 6 times repetition of the pericentromeric region of chromosome 18, which as a consequence must have been generated though a complex chain of multiple events. We propose a model as shown in figure 5. Our hypothesis is that the generation of the ring chromosome 18 may have been initiated by a transverse misdivision of the centromere or by successive breaks at or adjacent to the centromere (leading to loss of 18p) and in the long arm of chromosome 18. Subsequently a small ring chromosome may have been generated through U-type sister chromatin exchanges [6,17,18]. Due to the small size of the duplicon nonhomologous end joining [19,20] seems a less likely explanation. After DNA synthesis a subsequent sister chromatid exchange lead to the formation of a dicentric ring chromosome, containing 4 times the pericentric region of 18q10-10q11.2. Intrastrand homologous recombination and subsequent homologous end joining may have generated a monocentric ring chromosome and a bicentric ring chromosome consisting of 3 times the pericentric region. To prevent the interference with cell division most centromeres of the supernumerary ring had to be inactivated. This may explain the loss of the monocentric ring in subsequent cell divisions. A final event in the formation of the ring chromosome with 6 copies of the pericentromeric region of chromosome 18 and 4 centromeres might have been the entire doubling of the ring via a mechanism of sister chromatid exchange (SCE) [21,22].

The ring chromosome generated in such a complex chain of events must contain 4 centromeres. We tried to confirm this using FISH probes (CEP18) for centromere 18. However, while normal signals were observed on the normal paternal and maternal chromosomes 18, no centromere 18 signals were found on the ring chromosome 18 (data not shown). Since cell division is likely to be hampered by multiple functional centromeres, most of the centromeres on the supernumerary ring were likely to be inactivated. However, since FISH did not reveal the presence of centromere 18 signals on the ring chromosome it is possible that the centromeres were actually lost by an unknown other mechanism of ringformation and that neocentromeres might have been generated [23].

To our knowledge, only few publications describe partial trisomies and tetrasomies for segments of chromosome 18 which resulted from the presence of marker chromosomes or small supernumerary chromosomes [6,17]. However, rings of chromosome 18 have been reported in a significant number of cases in which the ring chromosomes replaced a normal maternal or paternal chromosome 18 [3-13]. In these cases most r(18) patients showed symptoms of 18q- or 18p- syndromes or a combination of these two[24]. Few cases of patients with supernumerary small ring chromosomes 18 have been described (table 1), but all cases had a structural organization and phenotype different from our case [5-7]. Our r(18) patient had anal atresia, abnormal lumbar and sacral vertebrae, hypospadia, cryptorchism, bifid scrotum, ureteral flow abnormality, radial dysplasia and thumb hypoplasia on the right upper extremity and preaxial polydactyly of the left hand. The patient described by Timur *et al*. with Klippel-Trenaunay Syndrome had a supernumerary r(18) chromosome that contained chromosomal material from 18p in 24% of his cells [5]. Callen et al. described a patient (case 9) who possessed a supernumerary r(18) chromosome in 85% of the cells, while the other cells showed a normal karyotype [6]. Unfortunately, it was unknown from which part of chromosome 18 the ring chromosome was derived. The patient had a short stature, normal intelligence and no dysmorphic features. Jenderny *et al*. described a female patient with a maternally derived small r(18) in all cells with breakpoints approximately at 18q11 and 18q23[7]. Unlike our patient, this patient did not possess a supernumerary ring chromosome 18. However, in the patient's mother the ring chromosome was present supernumerary resulting in a 47,XX,+r(18)[2]/46,XX[98] karyotype. The mother was phenotypically and mentally normal and there was no heart disease in the family. The relatively mild phenotypic characteristics of the mother may be due to the low percentage of mosaicism (2%). The latter case illustrates that there is also a potential risk for our patient to pass the supernumerary ring chromosome on to the next generation in a nonmosaic manner, possibly leading to congenital abnormalities.

**Table 1:** Clinical characterstics of the patient compared with other cases with supernumerary ring chromosomes 18 and other abnormal chromosomes 18

	Our case	[7]**mother**	[6]**case 9**	[5]	[28]	[29]**case 18-2**	[30]	[31]**child 3**	[32]
	patients with supernumerary ring chromosomes 18				patients with abnormal chromosomes 18				
Described karyotype	47,XY,+r(18)(q10q11.2)	47,XX,+r(18)(p1?1q2?3)[2]/46,XX[98]	47,XX,+r(18)[85%]/46,XX[15%]	mos 47,XY,+r(18)[13]/46,XY[42]	46,XX,dic(18;18)(q11.2;q21.3)	47,XX,min(18)(:p11.1q11.2)	mos +der(18)(p11.21q11.2)[68%]	ins(18)(p13.2;q12.2q11.2)	47,+min(18)(:p11.21q11.2:)[68%]/46[32%]
Birth weight	2670 g		2500 g	2948 g	TOP 22wks 520 g				
Developmental delay/mental retardation	-	-	-	+			possibly	-	possibly
Dysmophic features	+	-	-	+		-	possibly	-	possibly
Hearth disease		-				+			
Atrial septal defect						+			
Single umbilical artery	+								
Anal artresia	+								
Dysplastic lumbar and sacral vertebrae	+								
Penoscrotal hypospadia	+								
Cryptorchism	+								
Bifid scrotum	+								
Vesico ureteral reflux	+								
Trabeculated bladder	+								
Radial dysplasia	+								
Thumb hypoplasia	+								
Preaxial polydactyly	+								
VACTERL association	+								
Pancreas fissum	+								
Hypertrophy of the lower limb				+					
Long tapering fingers				+					
Elongated, thin feet				+					
Vascular malformations				+					
Splenomegaly				+					
Size discrepancy kidneys				+					
Renal arteriovenous malformations				+					
Hydronephrosis				+					
Esophageal varices				+					
Short stature			+						
Unilateral cleft lip and palate					+				
Anotia with atresia					+				
Low set ear					+				
Micorgnathia					+				
Rocker-bottom feet					+				
Left index finger crossed over 3^rd ^and 4^th ^fingers					+				
2^nd ^toe crossed over 3^rd ^toe					+				
Open ductus botalli					+	+			

To explain the phenotype of the patient by the aberrant expression of genes present on the ring chromosome is difficult. The large number of genes (~25 RefSeq genes) present in the chromosomal region of 18q10 - 18q11.2, including the OMIM disease genes RBBP8, NPC1, LAMA3, makes it complex to pinpoint the relative contribution of each gene to the phenotype. Furthermore, disease phenotypes are in most cases linked to deletions, while our patient presented with an octasomy of the particular genomic region.

## Conclusions

We described the *de novo *occurrence of a unique supernumerary ring chromosome leading to a partial octasomy of chromosome 18 in 11-13% of the peripheral blood lymphocytes of an adult male patient with phenotypic aberrations. The present case clearly illustrates the potential complexity of chromosomal aberrations in patients with phenotypic abnormalities.

## Methods

### Karyotyping

All studies were conducted with approval of the institutional review board at Erasmus MC Rotterdam. Informed consent was given and the study was performed according to the tenets of the Declaration of Helsinki. GTG-banded metaphases obtained from peripheral blood and fibroblast cultures were karyotyped using standard procedures. Karyotypes were obtained from the patient and both parents. Results were described in accordance with the ISCN 2009[25].

### Fluorescence in situ hybridization (FISH)

For FISH analysis of the ring chromosome the BAC clones RP11-79F3 (18q11.2) and RP11-411B10 (18p11.21) were selected from the University of California Santa Cruz (UCSC) http://genome.ucsc.edu/[26,27] genome browser (University of California, Santa Cruz, CA). Ten micrograms of DNA was isolated using an AutoGenPrep 3000 robot (Autogen, Holliston, MA) and after whole genome amplification (WGA, Repli-G) (Qiagen, Venlo, The Netherlands), the DNA was digested and labeled (Random Prime labeling system) (Invitrogen/Life Technologies, Carlsbad, CA) with Bio-16-dUTP or Dig-11-dUTP (Roche, Almere, The Netherlands). The FISH experiments were performed in duplicate according to standard protocols with minor modifications. The FISH probes were validated on control metaphase spreads. The whole chromosome 18 paint probe (wcp18) (Euro-Diagnostica, Malmö, Sweden) was used according to the same FISH protocol. FISH slides were analyzed with an Axioplan 2 Imaging microscope (Carl Zeiss, Sliedrecht, The Netherlands) and images were captured using Isis software (MetaSystems, Altlussheim, Germany).

### Array whole genome analysis

Genomic DNA was extracted from peripheral blood (Qiagen). DNA quantity (20-80 μg in 20 μL) was measured with a spectrophotometer (model ND-1000) (NanoDrop Technologies, Wilmington, DE), and quality was assayed on a bioanalyzer (model 2100) (Agilent, Palo Alto, CA). WGA was performed according to the manufacturer's instructions. The array platforms used were the single nucleotide polymorphism (SNP) microarray Affymetrix 250 K Nsp1 (Affymetrix, Santa Clara, CA) and the Illumina 610 quad arrays (Illumina, San Diego, CA). Copy number data was analyzed using Beadstudio software (Illumina) and Nexus software (Nexus BioDiscovery, El Segundo, CA).

### DNA-analysis

DNA-analysis by MLPA and direct sequencing of the coding regions of the genes SALL1, SALL4 en TBX5 of the patient showed no pathogenic abnormalities.

### GeneScan microsatellite markers

DNA was acquired from peripheral blood samples of the patient and both parents. Eight microsatellite markers were selected from the UCSC genome browser for identification of the parental origin of the ring chromosome: D18S1149, D18S1104, D18S869, D18S480, D18S1108, D18S1107, D18S66, and D18S819. For all microsatellites the same PCR program was used. After the initial denaturation (10 min, 95°C), 30 cycles were performed for denaturation (45 sec, 95°C), annealing (45 sec, 56°C), and elongation (1 min 30 sec, 72°C), followed by an extra elongation period of 10 min (72°C). PCR product sizes were determined using an ABI 3730xl capillary sequencer (Applied Biosystems/Life Technologies, Carlsbad, CA). Genotyping of the microsatellite markers was performed using GeneMarker software (SoftGenetics, State College, PA).

## Consent

Written informed consent was obtained from the patient for publication of this case report. A copy of the written consent is available for review by the Editor-in-Chief of this journal.

## Competing interests

The authors declare that they have no competing interests.

## Authors' contributions

LV designed research, performed research, analyzed data and wrote the paper. MD designed research, performed research, analyzed data. HD, JB and RG performed research. AH wrote the paper. PP designed research and analyzed data. AK designed research, analyzed data and wrote the paper. All authors read and approved the final manuscript.

## References

[B1] Cody JD Ghidoni PD DuPont BR Hale DE Hilsenbeck SG Stratton RF Hoffman DS Muller S Schaub RL Leach RJ Kaye CI Congenital anomalies and anthropometry of 42 individuals with deletions of chromosome 18q Am J Med Genet 1999 85 455 462 10.1002/(SICI)1096-8628(19990827)85:5<455::AID-AJMG5>3.0.CO;2-Z 10405442

[B2] Stankiewicz P Brozek I Helias-Rodzewicz Z Wierzba J Pilch J Bocian E Balcerska A Wozniak A Kardas I Wirth J Clinical and molecular-cytogenetic studies in seven patients with ring chromosome 18 Am J Med Genet 2001 101 226 239 10.1002/1096-8628(20010701)101:3<226::AID-AJMG1349>3.0.CO;2-# 11424138

[B3] Baumer A Giovannucci Uzielli ML Guarducci S Lapi E Rothlisberger B Schinzel A Meiotic origin of two ring chromosomes 18 in a girl with developmental delay Am J Med Genet 2002 113 101 104 10.1002/ajmg.10700 12400074

[B4] Miller K Pabst B Ritter H Nurnberg P Siebert R Schmidtke J Arslan-Kirchner M Chromosome 18 replaced by two ring chromosomes of chromosome 18 origin Hum Genet 2003 112 343 347 12574939 10.1007/s00439-002-0885-1

[B5] Timur AA Sadgephour A Graf M Schwartz S Libby ED Driscoll DJ Wang Q Identification and molecular characterization of a de novo supernumerary ring chromosome 18 in a patient with Klippel-Trenaunay syndrome Ann Hum Genet 2004 68 353 361 10.1046/j.1529-8817.2004.00095.x 15225160

[B6] Callen DF Eyre HJ Ringenbergs ML Freemantle CJ Woodroffe P Haan EA Chromosomal origin of small ring marker chromosomes in man: characterization by molecular genetics Am J Hum Genet 1991 48 769 782 2014800 PMC1682952

[B7] Jenderny J Caliebe A Beyer C Grote W Transmission of a ring chromosome 18 from a mother with 46,XX/47,XX, + r(18) mosaicism to her daughter, resulting in a 46,XX,r(18) karyotype J Med Genet 1993 30 964 965 10.1136/jmg.30.11.964 8301656 PMC1016610

[B8] Kosztolanyi G Mehes K Hook EB Inherited ring chromosomes: an analysis of published cases Hum Genet 1991 87 320 324 10.1007/BF00200912 1864607

[B9] Speevak MD Smart C Unwin L Bell M Farrell SA Molecular characterization of an inherited ring (19) demonstrating ring opening Am J Med Genet A 2003 121A 141 145 10.1002/ajmg.a.20184 12910493

[B10] Martin RH Cytogenetic determinants of male fertility Hum Reprod Update 2008 14 379 390 10.1093/humupd/dmn017 18535003 PMC2423221

[B11] Niikawa N Kajii T The origin of mosaic Down syndrome: four cases with chromosome markers Am J Hum Genet 1984 36 123 130 6230008 PMC1684390

[B12] Dagna Bricarelli F Pierluigi M Grasso M Strigini P Perroni L Origin of extra chromosome 21 in 343 families: cytogenetic and molecular approaches Am J Med Genet Suppl 1990 7 129 132 10.1002/ajmg.1320370726 1981472

[B13] Daniel A Malafiej P A series of supernumerary small ring marker autosomes identified by FISH with chromosome probe arrays and literature review excluding chromosome 15 Am J Med Genet A 2003 117A 212 222 10.1002/ajmg.a.10100 12599184

[B14] Daniel A Wu Z Bennetts B Slater H Osborn R Jackson J Pupko V Nelson J Watson G Cooke-Yarborough C Loo C Karyotype, phenotype and parental origin in 19 cases of triploidy Prenat Diagn 2001 21 1034 1048 10.1002/pd.164 11746161

[B15] Litzman J Brysova V Gaillyova R Thon V Pijackova A Michalova K Zemanova Z Lokaj J Agammaglobulinaemia in a girl with a mosaic of ring 18 chromosome J Paediatr Child Health 1998 34 92 94 10.1046/j.1440-1754.1998.00162.x 9568951

[B16] Los FJ van den Berg C Braat PG Cha'ban FK Kros JM Van Opstal D Ring chromosome 18 in a fetus with only facial anomalies Am J Med Genet 1996 66 216 220 10.1002/(SICI)1096-8628(19961211)66:2<216::AID-AJMG18>3.0.CO;2-W 8958334

[B17] Blennow E Nielsen KB Telenius H Carter NP Kristoffersson U Holmberg E Gillberg C Nordenskjold M Fifty probands with extra structurally abnormal chromosomes characterized by fluorescence in situ hybridization Am J Med Genet 1995 55 85 94 10.1002/ajmg.1320550122 7702104

[B18] Wik Sjostedt A Alatalo M Wahlstrom J von Dobeln U Olegard R Replication error, a new hypothesis to explain the origin of a supernumerary marker chromosome in a mentally retarded boy Hereditas 1989 111 115 123 10.1111/j.1601-5223.1989.tb00385.x 2625404

[B19] Schinzel A Kotzot D Brecevic L Robinson WP Dutly F Dauwerse H Binkert F Baumer A Ausserer B Trisomy first, translocation second, uniparental disomy and partial trisomy third: a new mechanism for complex chromosomal aneuploidy Eur J Hum Genet 1997 5 308 314 9412788

[B20] Bailey SM Goodwin EH DNA and telomeres: beginnings and endings Cytogenet Genome Res 2004 104 109 115 10.1159/000077474 15162023

[B21] Ieshima A Ogasawara N Yamamoto Y Kuroki Y A case of r(21) with stigmata of atypical Down syndrome Hum Genet 1980 55 65 69 10.1007/BF00329128 6450156

[B22] McGinniss MJ Kazazian HH Stetten G Petersen MB Boman H Engel E Greenberg F Hertz JM Johnson A Laca Z Mechanisms of ring chromosome formation in 11 cases of human ring chromosome 21 Am J Hum Genet 1992 50 15 28 1346075 PMC1682523

[B23] Choo KH Domain organization at the centromere and neocentromere Dev Cell 2001 1 165 177 10.1016/S1534-5807(01)00028-4 11702777

[B24] Schinzel A Catalogue of unbalanced chromosome aberrations in man 2001 Berlin, New York: Walter de Gruyter

[B25] Shaffer MLS LG Campbell LJ (eds) ISCN 2009: An International System for Human Cytogenetic Nomenclature, S. Karger, Basel, Switzerland 2009

[B26] Kent WJ Sugnet CW Furey TS Roskin KM Pringle TH Zahler AM Haussler D The human genome browser at UCSC Genome Res 2002 12 996 1006 12045153 10.1101/gr.229102 PMC186604

[B27] Karolchik D Kuhn RM Baertsch R Barber GP Clawson H Diekhans M Giardine B Harte RA Hinrichs AS Hsu F The UCSC Genome Browser Database: 2008 update Nucleic Acids Res 2008 36 D773 779 10.1093/nar/gkm966 18086701 PMC2238835

[B28] Lin CC Li YC Liu PP Hsieh LJ Cheng YM Teng RH Shi SL Tsai FJ Identification and characterization of a new type of asymmetrical dicentric chromosome derived from a single maternal chromosome 18 Cytogenet Genome Res 2007 119 291 296 10.1159/000112076 18253044

[B29] Liehr T Mrasek K Weise A Dufke A Rodriguez L Martinez Guardia N Sanchis A Vermeesch JR Ramel C Polityko A Small supernumerary marker chromosomes--progress towards a genotype-phenotype correlation Cytogenet Genome Res 2006 112 23 34 10.1159/000087510 16276087

[B30] Ballif BC Hornor SA Sulpizio SG Lloyd RM Minier SL Rorem EA Theisen A Bejjani BA Shaffer LG Development of a high-density pericentromeric region BAC clone set for the detection and characterization of small supernumerary marker chromosomes by array CGH Genet Med 2007 9 150 162 10.1097/GIM.0b013e3180312087 17413419

[B31] Starke H Senger G Kossakiewicz M Tittelbach H Rau D Rubtsov N Trifonov V Heller A Hartmann I Claussen U Liehr T Maternal insertion of 18q11.2-q12.2 in 18p11.3 of the same chromosome analysed by microdissection and multicolour banding (MCB) Prenat Diagn 2001 21 1049 1052 10.1002/pd.192 11746162

[B32] Ballif BC Rorem EA Sundin K Lincicum M Gaskin S Coppinger J Kashork CD Shaffer LG Bejjani BA Detection of low-level mosaicism by array CGH in routine diagnostic specimens Am J Med Genet A 2006 140 2757 2767 17103431 10.1002/ajmg.a.31539

